# A Preliminary Study on the Synthesis and Characterization of Multilayered Ag/Co Magnetic Nanowires Fabricated via the Electrodeposition Method

**DOI:** 10.1155/2013/837048

**Published:** 2013-08-29

**Authors:** Cheng-Hsiung Peng, Tsung-Yung Wu, Chyi-Ching Hwang

**Affiliations:** ^1^Department of Chemical and Materials Engineering, Ming Hsin University of Science and Technology, Hsinfeng, Hsinchu 304, Taiwan; ^2^School of Defense Science, Chung Cheng Institute Technology, National Defense University, Daxi, Taoyuan 335, Taiwan; ^3^Wapeon System Center, Chung Cheng Institute Technology, National Defense University, Daxi, Taoyuan 335, Taiwan

## Abstract

A single-bath electrodeposition method was developed to integrate multilayer Ag/Co nanowires with a commercial anodic alumina oxide (AAO) template with a pore diameter of 100–200 nm. An electrolyte system containing silver nitride and cobalt sulfide was studied using cyclic voltammetry, and the electrodeposition rate was varied to optimize the electrodeposition conditions. A constant stepwise potential and a variable cation ratio of [Co^2+^]/[Ag^+^] were used during electrodeposition. After the dissolution of the template in aqueous NaOH solution, multilayered Ag/Co nanowires were obtained with a composition of [Co]/[Ag_80_Co_20_], as identified by XRD and TEM, when [Co^2+^]/[Ag^+^] = 150. By annealing at 200°C for 1 h, uniformly structured (Co_99.57_/Ag_100_) nanowires were obtained. Compared with pure Co nanowires, the magnetic hysteresis loops showed a greater magnetic anisotropy for (Co_99.57_/Ag_100_) nanowires than for pure Co nanowires, corresponding to a change in the easy axis upon magnetization.

## 1. Introduction

The synthesis and study of nanoscale materials have attracted much attention in recent years. One-dimensional nanostructures, including nanowires, nanorods, and nanotubes, have many amazing properties such as high density, high aspect ratio, and low threshold voltage in field emission. On the other hand, the application of the giant magnetoresistance (GMR) effect found in 2-D metallic multilayers [1] has also been rigorously investigated for applications in the magnetic industry such as information storage and magnetic sensors [2, 3]. The development of high-density perpendicular magnetic recording encourages the trend to investigate new types of magnetic structures as the medium. With the combination of nanowires and multilayered structures, multilayered nanowires will exhibit special characteristics compared to conventional magnetic materials.

In general, a hard template containing nanometer-sized cylindrical pores is used as the membrane for the synthesis of magnetic nanowires and the pores are filled with segments of nanowires of different elements. Electrochemical synthesis utilizing the multibath or single-bath method is usually used to deposit the multimetal segments into the template for better efficiency and cheaper processing [4]. The most widely used templates for template-synthesized metallic nanowire arrays [5] are ion track-etched polycarbonate [6], anodic aluminum oxide (AAO) [7, 8], and mica [9]. Multibath electrodeposition was not considered in this research due to the difficulty of removing residual electrolyte containment from the nanometer channels [10, 11]. Recently, some studies on magnetic multilayered nanowires, including Co/Cu [12–14], Cu/Ni [15], Au/Co [16], and Ag/Co [17], used the single-bath electrodeposition technique, where two types of metallic ions coexist in the electrolyte during the deposition.

Due to the immiscibility of Ag and Co, an Ag/Co multilayer structure [18, 19] should possess a more distinguished interface than other sets of multilayer magnets, and it might be applicable in high-density perpendicular magnetic recording media. Therefore, in this research, a new electrolyte combined with a stepped-potential controlled system was developed for the synthesis of Ag/Co multilayered nanowires using the single-bath method. The electrochemical behavior and magnetic hysteresis of the nanowires are discussed below. 

## 2. Experimental Methods

**Figure 1:**
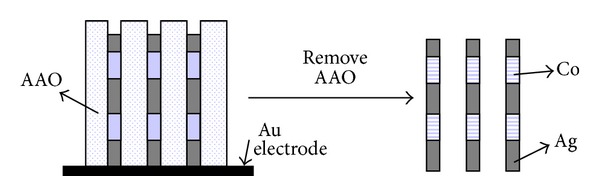
Multilayered nanowires structure.

To obtain multilayer nanowires, it was necessary to electrodeposit the metal segments within the channels of the AAO in sequence. After dissolving the template in NaOH, a multilayer structure was obtained, as shown schematically in Figure 1. Commercially available nanoporous alumina membranes (AAO) with a thickness of 60 *μ*m and a nominal pore diameter of 100 nm (actual range: 100 nm~200 nm) were used. The pore density of the AAO was 1 × 10^10^, and the pore interdistance was approximately 50–70 nm, which was confirmed by SEM analysis. The electrolyte used in the experiment contained CH_3_COONH_4_, AgNO_3_, and CoSO_4_ · 7H_2_O developed by our group. The major reactions are listed below:

(1) $$\begin{array}{c} \text{Ag}={\text{Ag}}^{+}+{\text{e}}^{-}\;\;E=-\;0.799\;\text{V}\\ \text{Co}={\text{Co}}^{2+}+2{\text{e}}^{-}\;\;E=0.277\;\text{V}. \end{array}$$

The solubility product of Ag(CH_3_COO), *K*_sp_ = 2.3 × 10^−29^, is much lower than that of Ag(NH_3_)^2+^ (*K*_sp_ = 6.3 × 10^−8^), so the results of precipitation during the first few minutes were observed closely when the binary-electrolyte system was prepared in CH_3_COONH_4_. Finally, the formation of complex ion Ag(NH_3_)^2+^ gradually substituted Ag(CH_3_COO) to form a clear electrolyte solution. 

The oxidation potentials and electrodeposition rate can be altered by altering the ion concentrations, such as Ag^+^ and Co^2+^, according to the Nernst equation and kinetic theory as follows:

(2) $$\begin{array}{c} E=E^{0}-\frac{2.3RT}{nF}\log Q, \end{array}$$

where *Q* is the reaction quotient, *R* is the gas constant (8.314 J mol^−1 ^K^−1^), *n* is mole number of electrons involved in the reaction, *F* is the Faraday constant (96485 C mol^−1^), and *T* is the temperature (in K). The cyclic-voltammetry and competing rate analysis of Ag/Co electrodeposition were used to refine and optimize the electrodeposition conditions for the single-bath electrodeposition. Cyclic-voltammetry was measured with a CHI 604A electrochemical analyzer (CH Instrument, USA) with a scanning rate of 0.1 V/sec. Glassy carbon and Pt wire were used as the working and counter electrodes, respectively, while Ag/AgCl was used as the reference electrode.

A power supply, such as a source-meter (Keithly 2400, Keithly USA), was used to control the parameters of each step, including apply voltage, and deposition time, during the multi-step electrodeposition using a PC-based program. In addition to the step electrodeposition method, the characteristics of the potential segments were also varied, such as decreasing the segment time and off-potential time to overcome the charging problem. Anodic aluminum oxide (AAO) was used as the template for deposition to accommodate the post annealing process. A DC-sputtered gold layer with a thickness of approximately 200 nm was used as the conducting bottom layer of the AAO. For the convenience of characterization, the AAO membrane was dissolved in 5% NaOH after electrodeposition. X-ray diffraction (XRD) and electron microscopy, such as field-emission scanning electron microscopy (FESEM) and transmission electron microscopy (TEM), were used to identify the compositions and structures of the nanowires. The magnetic behavior of the Ag/Co multilayered nanowires within AAO was also investigated with a vibrating sample magnetometer VSM.

## 3. Results and Analysis

### 3.1. Electrochemical Analysis

**Figure 2:**
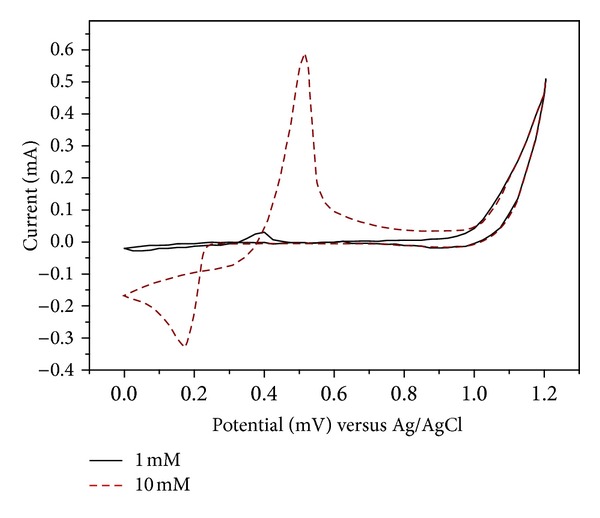
Cyclic-voltammetry analysis by altering [Ag^+^] with constant [Co^2+^] = 0.15 M.

**Figure 3:**
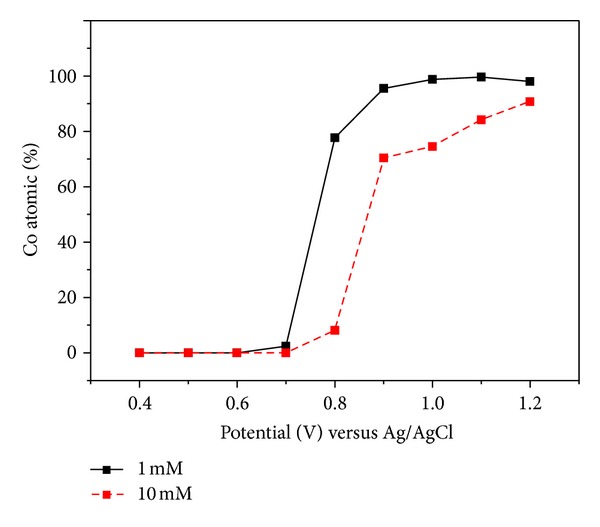
Electrodeposition rate upon altering [Ag^+^] with constant [Co^2+^] = 0.15 M.

**Figure 4:**
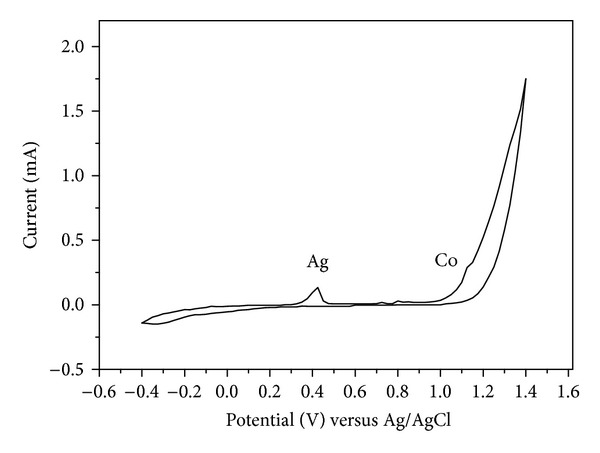
Cyclic-voltammetry analysis of the solution with [Co^2+^]/[Ag^+^] = 150.

Figure 2 shows the cyclic-voltammetry analysis by altering [Ag^+^] with constant [Co^2+^] = 0.15 M. It was observed that the concentration of [Ag^+^] played a more important role than that of [Co^2+^]. The difference in the electrodeposition rate at various voltages was examined by the energy-dispersive spectroscopy (EDS) analysis of the deposition layer, and the results are shown in Figure 3. With two different [Ag^+^] equal to 1 mM and 10 mM, the deposition of the Co layer did not start until 0.7 V, and the stacking rate of Ag and Co increased drastically with increasing voltage. When the applied voltage was high enough to force the reduction of Co^2+^ ions, the Co/Ag ratio increased. However, the electrolysis of water also occurred at a higher voltage to break the competition balance of the ions, and the effective current density decreased. Therefore, the reduction of the two metal ions decreased. As a result, a nearly pure Co layer (Co = 98.83%, Ag = 1.07%) was produced when the applied voltage was 1.1 V versus Ag/AgCl at the concentration ratio Co/Ag = 150. In the cyclic-voltammetry analysis shown in Figure 4, the ion concentration ratio between [Co^+^] and [Ag^2+^] was fixed at Co/Ag = 150. It was observed that the reduction of Ag^+^ began at about 0.3 V (versus Ag/AgCl), and Co did not begin to appear until 0.8 V.

Although pure Ag segments could be obtained when the applied voltage was below 0.6 V, the deposition rate was too slow to complete the experiments within an acceptable time. In addition, the reduction effect could also strongly dissolve the cobalt layer deposited previously. Therefore, two factors were considered in the design of the deposition sequence of Co and Ag: the applied potential and the deposition time. The Co layer was deposited at 1.0 V, and the Ag layer was deposited at 0.65 V, which is close to the upper limit for Co reduction. The times were Co: 50 s, Ag: 100 s, and off-potential time: 30 s.

Within the nanoscale channel, an immediate change in potential within a few seconds was accompanied by problems such as charging resulting from the electric double layer and concentration unbalance of the two metal ions near to the interface of the metal deposition layer and electrolyte. Applying a stair-shape periodical potential design like that in Figure 1 effectively prevents microbubbles and over electrolyzing. 

### 3.2. Structure Analysis

**Figure 5:**
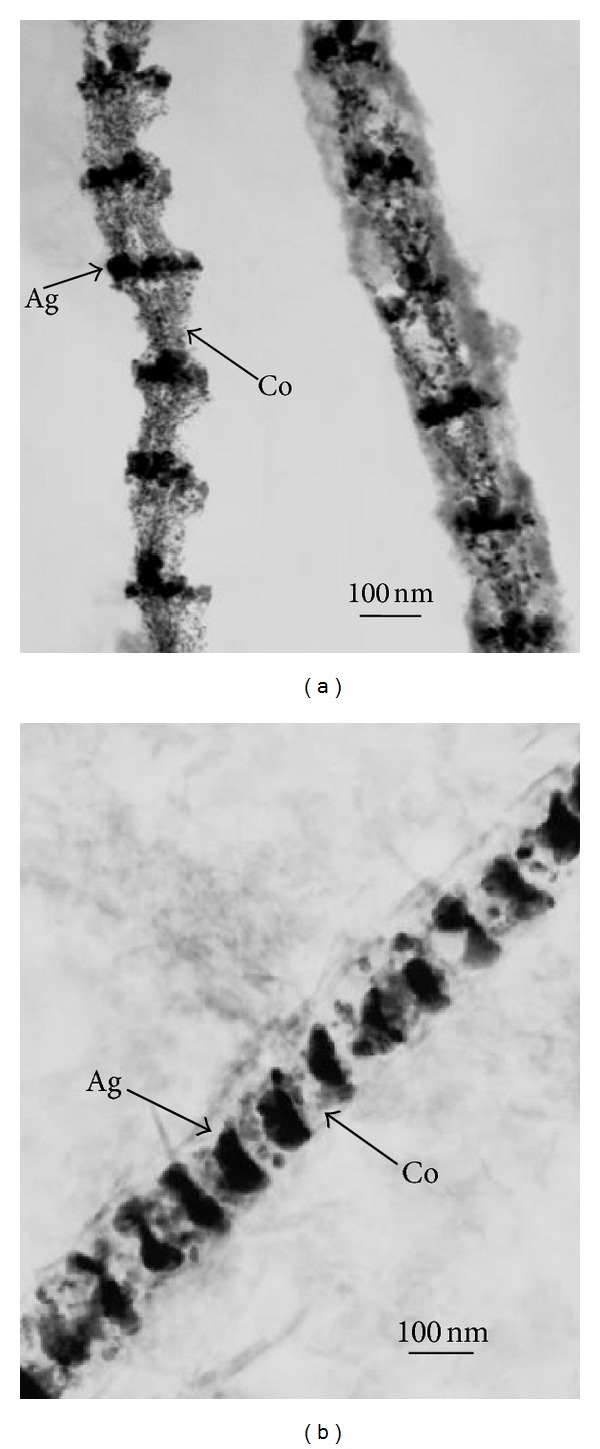
(a) TEM bright-field image of [Co]/[Ag_80_Co_20_] multilayered nanowires, and (b) Co_99.57_/Ag_100_ multilayered nanowires after annealing at 200°C for 1 h.

The optimized deposition conditions and program setting are given in Table 1. The Ag and Co segments were deposited in the AAO template with 30 rounds. After dissolving the AAO template in 5% NaOH solution for 30 min, the nanowires were imaged, and the micrographs are shown in Figure 5. The TEM bright field image in Figure 5(a) shows a clear image contrast between two nearby segments, which represents the Ag and Co layers. The darker, shorter part was verified to contain mainly Ag. Both diffraction patterns and TEM-EDS were used to identify and confirm the composition of the segment along the nanowire axis. The multilayered nanowires were composed of two types of segments, [Co] and [Ag_80_Co_20_], and developed a homogenous and period structure. Moreover, an indistinct interface can be clearly observed in the TEM bright image. This result can be explained by the original stage-potential design and the appearance of the Co-Ag^+^ interface redox reaction. To obtain a clear interface between the two metals, they were annealed at 200°C for 1 hr.  Figure 5(b) shows the TEM image after annealing, revealing better and clearer segments of Co_99.57_/Ag_100_ multilayered nanowires. 

### 3.3. Magnetic Analysis

**Figure 6:**
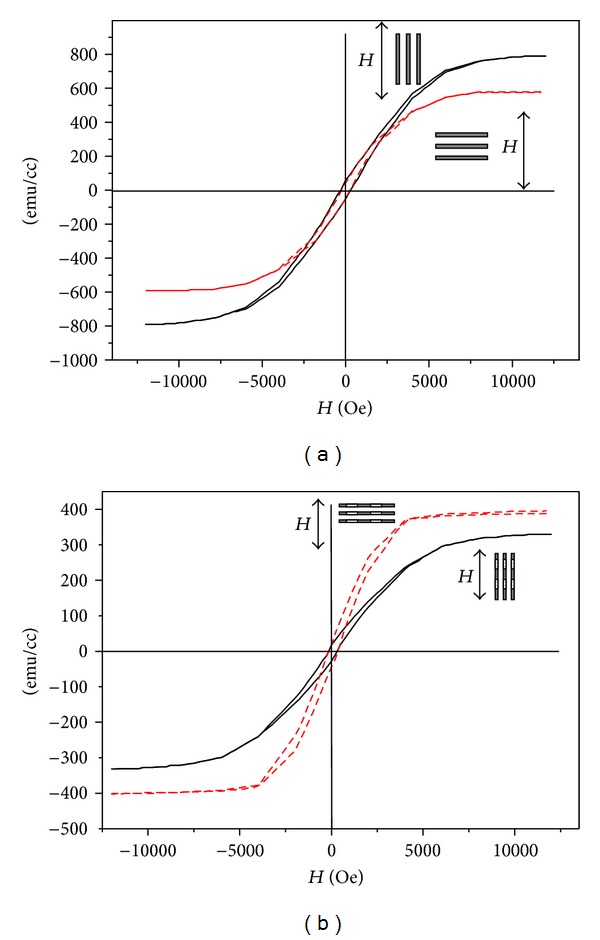
(a) Magnetic hysteresis of pure Co nanowires and (b) Co_99.57_/Ag_100_ multilayered nanowires along two vertical axes.

For storage applications, it is interesting to compare the magnetic hysteresis loops of pure Co nanowires and Ag/Co multilayered nanowires. The hysteresis loop measurements were performed with the magnetic field parallel and perpendicular to the nanowire axis, as shown in Figures 6(a) and 6(b), respectively. The pure Co nanowires had a *H*_*c*_ of 300 Oe when the applied magnetic field was parallel to the long axis of the nanowires, compared to a *H*_*c*_ of 225 Oe with a perpendicular field. The Ag/Co multilayer nanowires had almost the same coercivity *H*_*c*_ of 210 Oe but showed obvious magnetic anisotropy and lower saturation magnetization (Ms) than the pure Co nanowires. The pure Co nanowires showed easier orientation along the axis (easy axis). However, the easy axis of the Ag/Co multilayered nanowires was oriented perpendicular to the axis of the nanowire. However, a more detailed characterization of electrodeposited Ag/Co multilayered nanowires is required to elucidate the magnetic behavior of the nanowires. 

## 4. Conclusion

In this study, electrochemical experiments and analyses were used to determine the optimized conditions to synthesize Ag/Co multilayer nanowires that were 100 nm in diameter by single-bath electrodeposition using an AAO template. The segment composition of the nanowires was [Co]/[Ag_80_Co_20_], which was not stable or homogeneous throughout the nanowire. However, nearly pure Co/Ag nanowires of Co_99.57_/Ag_100_ could be obtained by annealing, and they showed different magnetic properties compared to pure Co nanowires, such as more obvious anisotropy and a change in the easy axis.

## Figures and Tables

**Table 1:** Deposition parameters of Ag/Co multilayered nanowires.

Ingredient	Concentration (M)	Potential (V)	Segment time (s)
CoSO_4_·7H_2_O	0.15	1.0	100
AgNO_3_	0.005	0.65	200
CH_3_COONH_4_	0.4	Off	Off time : 30

## References

[1] Baibich MN, Broto JM, Fert A (1988). Giant magnetoresistance of (001)Fe/(001)Cr magnetic superlattices. *Physical Review Letters*.

[2] Valet T, Fert A (1993). Theory of the perpendicular magnetoresistance in magnetic multilayers. *Physical Review B*.

[3] Piraux L, George JM, Despres JF (1994). Giant magnetoresistance in magnetic multilayered nanowires. *Applied Physics Letters*.

[4] Schwarzacher W, Lashmore DS (1996). Giant magnetoresistance in electrodeposited films. *IEEE Transactions on Magnetics*.

[5] Martin CR (1994). Nanomaterials: a membrane-based synthetic approach. *Science*.

[6] Ounadjela K, Ferré R, Louail L (1997). Magnetization reversal in cobalt and nickel electrodeposited nanowires. *Journal of Applied Physics*.

[7] Aimawiawi D, Coombs N, Moskovits J M (1991). Magnetic properties of Fe deposited into anodic aluminum oxide pores as a function of particle size. *Journal of Applied Physics*.

[8] Schwanbeck H, Schmidt U (2000). Preparation and characterization of magnetic nanostructures using filtration membranes. *Electrochimica Acta*.

[9] Sun L, Chien CL, Searson PC (2000). Fabrication of nanoporous single crystal mica templates for electrochemical deposition of nanowire arrays. *Journal of Materials Science*.

[10] Leisner P, Nielsen CB, Tang PT, Dorge TC, Moller P (1996). Methods for electrodepositing composition-modulated alloys. *Journal of Materials Processing Technology*.

[11] Bradley PE, Landolt D (1999). Pulse-plating of copper-cobalt alloys. *Electrochimica Acta*.

[12] Blondel A, Doudin B, Ansermet J-P (1997). Comparative study of the magnetoresistance of electrodeposited Co/Cu multilayered nanowires made by single and dual bath techniques. *Journal of Magnetism and Magnetic Materials*.

[13] Gómez E, Labarta A, Llorente A, Vallés E (2002). Characterisation of cobalt/copper multilayers obtained by electrodeposition. *Surface and Coatings Technology*.

[14] Tian M, Wang J, Kurtz J, Mallouk TE, Chan MHW (2003). Electrochemical growth of single-crystal metal nanowires via a two-dimensional nucleation and growth mechanism. *Nano Letters*.

[15] Guo Y-G, Wan L-J, Zhu C-F, Yang D-L, Chen D-M, Bai C-L (2003). Ordered Ni-Cu nanowire array with enhanced coercivity. *Chemistry of Materials*.

[16] Valizadeh S, Hultman L, George J-M, Leisner P (2003). Template synthesis of Au/Co multilayered nanowires by electrochemical deposition. *Advanced Functional Materials*.

[17] Valizadeh S, George JM, Leisner P, Hultman L (2002). Electrochemical synthesis of Ag/Co multilayered nanowires in porous polycarbonate membranes. *Thin Solid Films*.

[18] Kenane S, Chainet E, Nguyen B, Kadri A, Benbrahim N, Voiron J (2002). Giant magnetoresistance in Co-Ag granular films prepared by electrodeposition. *Electrochemistry Communications*.

[19] Valizadeh S, Holmbom G, Leisner P (1998). Electrodeposition of cobalt-silver multilayers. *Surface and Coatings Technology*.

